# Partnering Parasites: Evidence of Synergism between Heavy *Schistosoma haematobium* and *Plasmodium* Species Infections in Kenyan Children

**DOI:** 10.1371/journal.pntd.0001723

**Published:** 2012-07-24

**Authors:** Lia S. Florey, Charles H. King, Melissa K. Van Dyke, Eric M. Muchiri, Peter L. Mungai, Peter A. Zimmerman, Mark L. Wilson

**Affiliations:** 1 Department of Epidemiology, University of Michigan, Ann Arbor, Michigan, United States of America; 2 Center for Global Health and Diseases, Case Western Reserve University, Cleveland, Ohio, United States of America; 3 Division of Vector Borne and Neglected Tropical Diseases, Ministry of Public Health and Sanitation, Nairobi, Kenya; 4 Msambweni Field Station, Msambweni, Kenya; London School of Hygiene & Tropical Medicine, United Kingdom

## Abstract

**Background:**

Residents of resource-poor tropical countries carry heavy burdens of concurrent parasitic infections, leading to high rates of morbidity and mortality. This study was undertaken to help identify the social and environmental determinants of multiple parasite infection in one such community.

**Methodology/Principal Findings:**

Residents of Kingwede, Kenya aged 8 years and older were tested for presence and intensity of *S. haematobium* and *Plasmodium* spp. infections in a cross-sectional, household-based, community survey. Using General Estimating Equation (GEE) models, social and environmental determinants associated with patterns of co-infection were identified, with age being one of the most important factors. Children had 9.3 times the odds of co-infection compared to adults (95%CI = 5.3–16.3). Even after controlling for age, socio-economic position, and other correlates of co-infection, intense concomitant infections with the two parasites were found to cluster in a subset of individuals: the odds of heavy vs. light *S. haematobium* infection increased with increasing *Plasmodium* infection intensity suggesting the importance of unmeasured biological factors in determining intensity of co-infection.

**Conclusions/Significance:**

Children in this community are more likely to be infected with multiple parasites than are adults and should therefore be targeted for prevention and control interventions. More importantly, heavy infections with multiple parasite species appear to cluster within a subset of individuals. Further studies focusing on these most vulnerable people are warranted.

## Author Summary

Parasitic diseases such as malaria and schistosomiasis account for a large proportion of global morbidity and mortality by contributing to malnutrition, developmental delays, loss of productivity, negative birth outcomes, disfigurement, physical handicap and social stigma. We studied these two infections in a community-wide, household-based study among residents of a coastal Kenyan village. With the use of newer PCR methods to detect sub-clinical malaria infections, and with the aid of statistical methods that could adjust for the household-level clustering of exposure factors, we found evidence that people of low socio-economic position are more likely to experience co-infection by these parasites. In addition, among children, we found that heavy infections clustered within a subset of individuals independent of their water contact, night activity, bednets use, and household distance to water, thus providing indirect evidence for biological interaction between these parasites in human hosts. If confirmed, these findings indicate that interventions focused on prevention and control of such co-infections could be concentrated on the most vulnerable groups of people in order to maximize benefits where resources are limited. Further studies of immunological and behavioral risk are warranted.

## Introduction

Parasitic infections account for a large proportion of the burden of disease in Kenya, with far-reaching effects on the nation's health and economy. Malaria, for example, represents 30–50% of all outpatient visits to health facilities, causing more than 20% of deaths in children less than 5 years old (yo) [1]. Neglected tropical diseases such as urinary schistosomiasis are important causes of morbidity in Kenya as well. Urinary schistosomiasis is estimated to affect roughly one-quarter of the Kenyan population leading to anemia, impaired growth, development and cognition [2] and other adverse outcomes. The parasites that cause these diseases are co-endemic in coastal Kenya subjecting the local population to a substantial, possibly synergistic disease burden [3].

Evidence exists that morbidities are likely to be compounded in individuals harboring multiple parasites, as compared to those with single infections. Co-infections with helminths and *Plasmodium* species, for example, increase various negative health effects, including organomegaly [4], low birth weight [5], and anemia [6], [7]. The morbidities associated with co-infections also are likely to depend on parasite loads [6], [8], [9] as seen in single-species *Plasmodium* infections [10], [11] and *S. haematobium* infections [12]. Understanding the complexities of the pathogen-host landscape in settings endemic for multiple human parasites is essential for mitigating morbidities. Identifying interspecies associations could advance intervention by targeting efforts that have the most prevention and treatment benefits. Despite extensive research on these individual infections, surprisingly little is known about the distributions, causes and effects of co-infections (polyparasitism).

At the population level, the distribution of polyparasitism depends on risk factors for individual infections and the extent to which these factors are shared across species. Factors known to influence the distribution of single species infection with *Plasmodium* spp. and *S. haematobium* include both social and environmental conditions. Some examples include poverty, access to safe water and sanitation, access to health care, water use behaviors and use of bednets [13]–[16]. Socioeconomic and ecologic parameters are sometimes difficult to separate as they often overlap in space. Co-infections may be spatially clustered if households are located near habitats suitable for transmission and if land use is socially and spatially patterned [17]–[19].

Alternatively, clustering of infections may be caused by biological synergism within the host. Evidence for this is mixed, however, with some studies showing increased susceptibility to clinical malaria in persons with helminth infection [20], [21], some showing protective effects of helminth infections [22] and others finding no effect [23], [24].

To improve understanding of causes and effects of polyparasitism, we undertook studies of infection by *Plasmodium* spp., and *Schistosoma haematobium* among members of a community in coastal south-eastern Kenya. The specific research objectives were to describe the prevalence and intensity of single and multiple species infections, identify individual and household-level correlates of single and multiple infections, and determine associations between infection prevalence and intensities after controlling for contextual effects.

## Methods

### Study site and population

**Figure 1:**
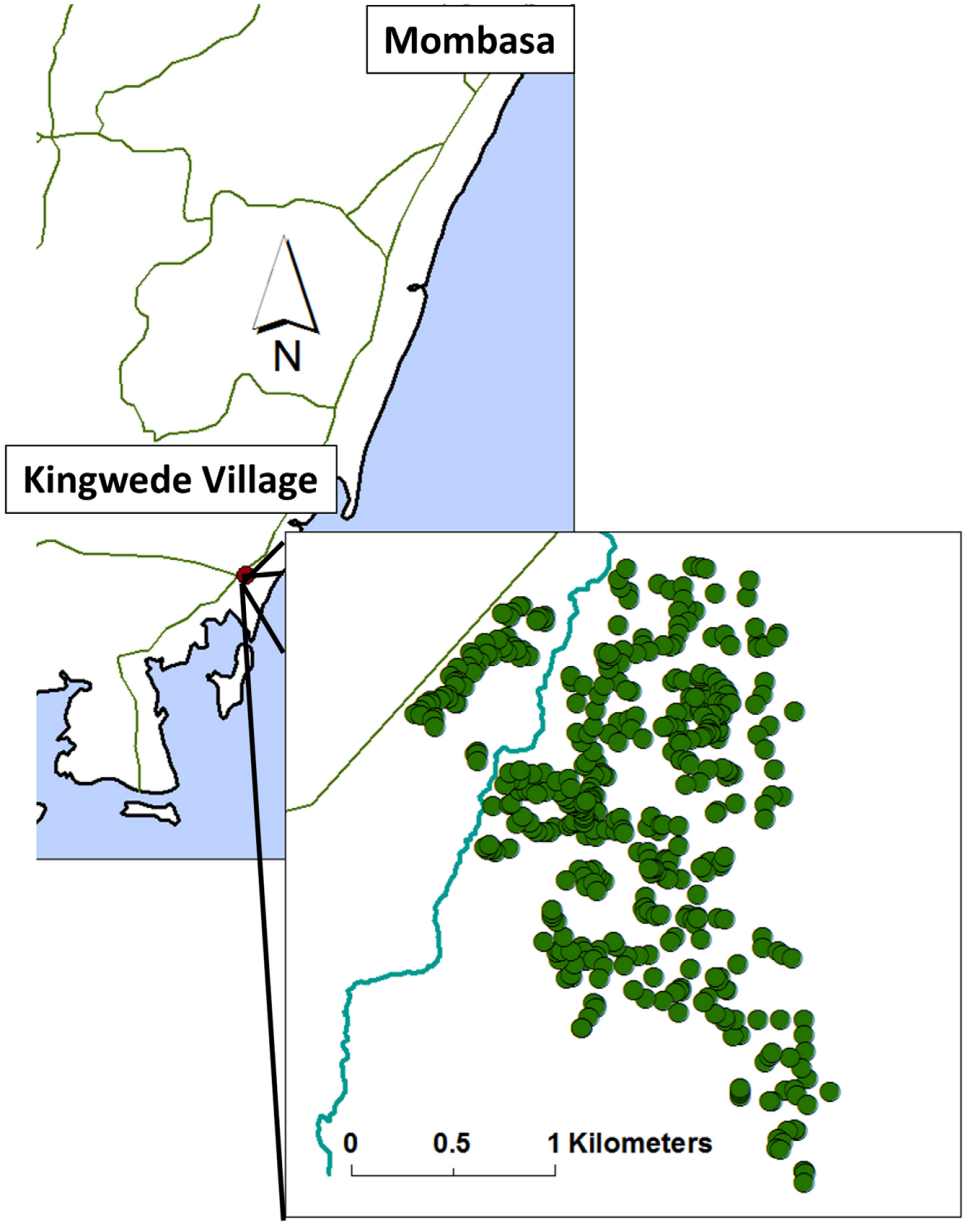
Map of study location. The location of Kingwede Village, 50 km southwest of Mombasa, in Coast Province, Kenya Inset shows the distribution of village households in relation to the main local stream and the highway to Mombasa.

For this cross-sectional study, a complete demographical profile of Kingwede, a rural village in coastal Kenya located ∼50 km southwest of Mombasa (see map, Figure 1), was compiled in December 2005. Trained interviewers identified every house in the study area and collected information (sex, age, relationship to household head, and years of residence) on each person who slept in that house the previous night. Subsequently, every individual ≥8 years old (yo) was invited to participate in the study during May through July 2006.

Kingwede residents are predominantly subsistence farmers and fishermen of the Digo tribe. Electricity and access to piped water are virtually nonexistent in the village. Pumped, potable water is available at 8 locations in the village. A seasonal stream and ponds are used for washing clothes, bathing and swimming. The study site is bordered on the northwest by the paved road to Tanzania, on the southeast by the Indian Ocean (3 km) and comprises a ∼6 sq. km area.

### Parasitological diagnosis and clinical information

Participants were asked to provide a midday urine sample on two consecutive days and one finger-prick blood sample. Presence of *S. haematobium* eggs was determined by microscopic examination of filtered midday urine. Two 10 ml aliquots of each stirred urine sample were filtered using 12-µm pore Nucleopore filters (Nucleopore, Pleasanton, CA) and mounted on microscope slides for examination [25]. Positive *S. haematobium* samples were defined as having ≥1 egg in at least one of the two samples.

Finger-prick blood (∼200 µL) that had been collected in potassium ethylene- diaminetetraacetic acid (K-EDTA)-coated Vacutainer tubes and stored at −20°C was further evaluated for presence and quantity of circulating parasites at Case Western Reserve University in Cleveland, OH. *Plasmodium* spp. was detected in these preparations using polymerase chain reaction/ligase detection reaction fluorescent microsphere-based assay (PCR/LDR-FMA) as previously described [26]–[28]. Briefly, DNA was extracted, amplified and added to a multiplex, species-specific ligase detection reaction (LDR) where species-specific primers hybridized to target sequences and were subsequently labeled with oligonucleotide probes with fluorescent capacity. A Bio-Plex array reader (Bio-Rad Laboratories, Hercules, CA) was used for detection of fluorescence in a species-specific manner. Samples were considered positive if they had a median fluorescent intensity (MFI)>2 standard deviations above those of negative controls (MFI above 205, 260, or 220 for *P. falciparum* (Pf), *P. malariae* (Pm) and *P. ovale* (Po), respectively). Of note, no *Plasmodium vivax* infection was detected in this study.

### Individual and Household Questionnaires

Once biological measures were obtained, trained interviewers conducted questionnaires with all willing participants. Different questionnaires were used for adults and for children. These questionnaires included queries aimed at assessing effect modifiers or confounders for infection status, including socio-economic position (SEP), knowledge, attitudes and practices (KAP) regarding malaria and schistosomiasis, mosquito avoidance behaviors, and water contact patterns. Household-level information (e.g. ownership of assets) was extracted from questionnaire responses of the household head or most senior household member, and was applied to other household members in analyses. All questionnaire data relied on self-reporting by participants or head-of-household responses. Other household-level information was collected by the interviewers who observed house quality (e.g. roofing and construction material, presence of window screens, etc.).

### Spatial data

The location of each house entrance (latitude, longitude) was measured using a handheld GPS device (Garmin eTrex Summit, Olathe, KS). A 1 km resolution IKONOS image of the study area was obtained from GeoEye (www.geoeye.com, Dulles, VA) for use in spatial and environmental analyses. ArcGIS v. 9.1 (ESRI, Redlands, CA, USA) was used to create spatial variables such as Euclidean distances from each house to water sources.

### Ethics

Following standard participant recruitment procedure used by other researchers in this area, prior to the community-wide parasitological survey, awareness of the study aims, risk, and potential benefits was raised through meetings with district and village leaders. These were followed by community-based, village-wide information meetings addressing concerns and questions. A village-wide demographic survey was then conducted to enumerate and code houses, and establish the age, sex, and number of individuals in each household. Written informed consent was obtained from adults and child assent accompanied by written parental consent was obtained for each child for participation in the parasitological survey and before administration of questionnaires. Approval for this study was granted by the University of Michigan Institutional Review Board (IRB# H05-00008982-I) and the Ethical Review Committee of the Kenya Medical Research Institute (KEMRI).

### Variable Management and Statistical Methods

Intensity of infection was defined as arithmetic mean eggs per 10 mL of the replicate urine filtrations for *S. haematobium*. For *Plasmodium* spp. infections, a standardized measure of MFI was created. All values below the aforementioned cut-off values were set to zero. Subsequently, the MFI were divided by the maximum observed value for a range of 0 to 1. This method was used separately for each *Plasmodium* species. A summed value was then created to represent burden of any *Plasmodium* species. The same method was used to create standardized *S. haematobium* intensity scores. Ordered categorical variables also were created based on the density distribution of each parasite. Intensities of each infection were divided into five, approximately equal categories based on natural cut-points in the distributions (0–4; no infection to heaviest). Finally, binary variables were created to define heavy infections as follows: *S. haematobium* ≥100 eggs per 10 mL urine [12], [29] (15% of individuals testing positive for *S. haematobium*); and *Plasmodium* spp. ≥0.55 standardized score (selected to include the two most heavily infected categories representing 26.9% of *Plasmodium*-infected individuals).

All data were double entered in Microsoft Access and analyzed using SAS 9.1 (SAS Institute, Inc., Cary, NC) and SPSS (v. 20, IBM SPSS, New York, NY) software. The outcome of co-infection was defined in several different ways for these analyses: A binary variable (Y/N) for *Plasmodium* spp. - *S. haematobium* co-infection was used to identify important covariates; a multinomial variable (no infection, *S. haematobium* only, *Plasmodium* spp. only, *S. haematobium* and *Plasmodium* spp.) was used to examine associations between parasites. For analyses of infection intensity outcome, variables included heavy *Plasmodium* spp. infection (Y/N) and heavy *S. haematobium* infection (Y/N). Covariates of primary interest in these analyses were age, sex, knowledge of malaria (MKAP), knowledge of schistosomiasis (SKAP), individual educational attainment, regular income (Y/N), use of bednets (Y/N), night outdoor activities, water contact behaviors, and recent malaria diagnosis. Household-level variables included household socio-economic position (SEP) and distance to the local stream. KAP questions (N = 3 for both malaria and schistosomiasis) were used to create a scale (0–3) for each infection based on the number of correct responses. Individual educational attainment was measured by highest class completed. Five categories of educational status were created based on quintiles of distributions. Education was ignored in subsequent analyses due to co-linearity with age in this study population. Bed net use was treated as a binary variable and did not distinguish between treated and untreated nets. A binary variable was created to measure effective treatment of malaria with medication in the past month. Water contact behaviors were assessed by combining responses to questionnaire data such that participants reporting swimming, fishing, bathing, washing dishes or washing clothes in water sources potentially infected with *S. haematobium* were considered exposed. Household SEP was assessed with an asset index constructed using principal components analysis of questionnaire responses [30], [31]. Assets measured include electricity, radio, television, bicycle, motor vehicle, land ownership, domestic animals and toilet. The score also included information on crowding, quality of house construction, and numbers of full and part time workers in a household. Missing data necessary for construction of the household SEP score were first imputed using the IVEware (Imputation and variance estimation software) SAS macro [32]. Each individual was assigned an SEP score corresponding to their household and this household SEP score was treated as a continuous variable.

General estimating equation (GEE) models with exchangeable correlation matrix structures and logistic distributions were used to estimate the association between odds of infection, both single and co-infection, and other social and environmental variables. Each infection outcome was assessed separately, thus in models of *Plasmodium* infection the referent group was individuals without *Plasmodium* infection regardless of *S. haematobium* infection status, and vice versa. In models of co-infection the referent group was individuals with single infections or no infections. This analytic approach accounts for household clustering in the data and estimates fixed effects of variables averaged across households. To identify individual and household-level variables associated with polyparasitism, all individual and household-level variables significantly associated with co-infection at α = 0.1 were included in multiple logistic GEE models. Although GEE models can be written for multinomial distributions using SAS 9.1, these models only accommodate ordinal outcome variables; thus associations with covariates assume a stepwise relationship between the infection categories. For simplicity in interpreting findings, we employed logistic GEE models for this section of the analyses. Backward elimination, in which the least significant effect as identified by Wald tests is removed from the model stepwise, was then used to select models best predicting the outcomes. Datasets for this analysis were limited to include only participants with complete biological and questionnaire data devoid of any missing responses. Continuous variables were centered on the grand mean.

Intensity of infection variables were similarly identified using logistic GEE models. In this case, models were restricted to infected individuals using a binary outcome of heavy versus light infection for each parasite and for co-infection. This was done in order to isolate factors related to the quantity of parasites an individual might carry from those influencing whether or not infection occurs.

To identify synergy or antagonism between infections, potential confounders, identified in the logistic GEE models of co-infection previously described, were included in multinomial GEE models. The outcome variable in these models was infection status with four potential values: 0 = uninfected; 1 = only *S. haematobium*; 2 = only *Plasmodium* spp.; 3 = co-infected. Co-infection was set as the referent category. Associations were considered significant at an α = 0.05 for the Type 3 likelihood ratio test of the co-infection regression coefficient in the full model, controlling for all other variables.

To examine associations between intense infections of the two species, logistic GEE models of heavy single infections included a variable for the intensity of the second infection, treated as an independent, ordered categorical variable (standardized intensity score) with 5 categories (0 to 4). These analyses were restricted to infected individuals.

## Results

### Characteristics of the study population

**Figure 2:**
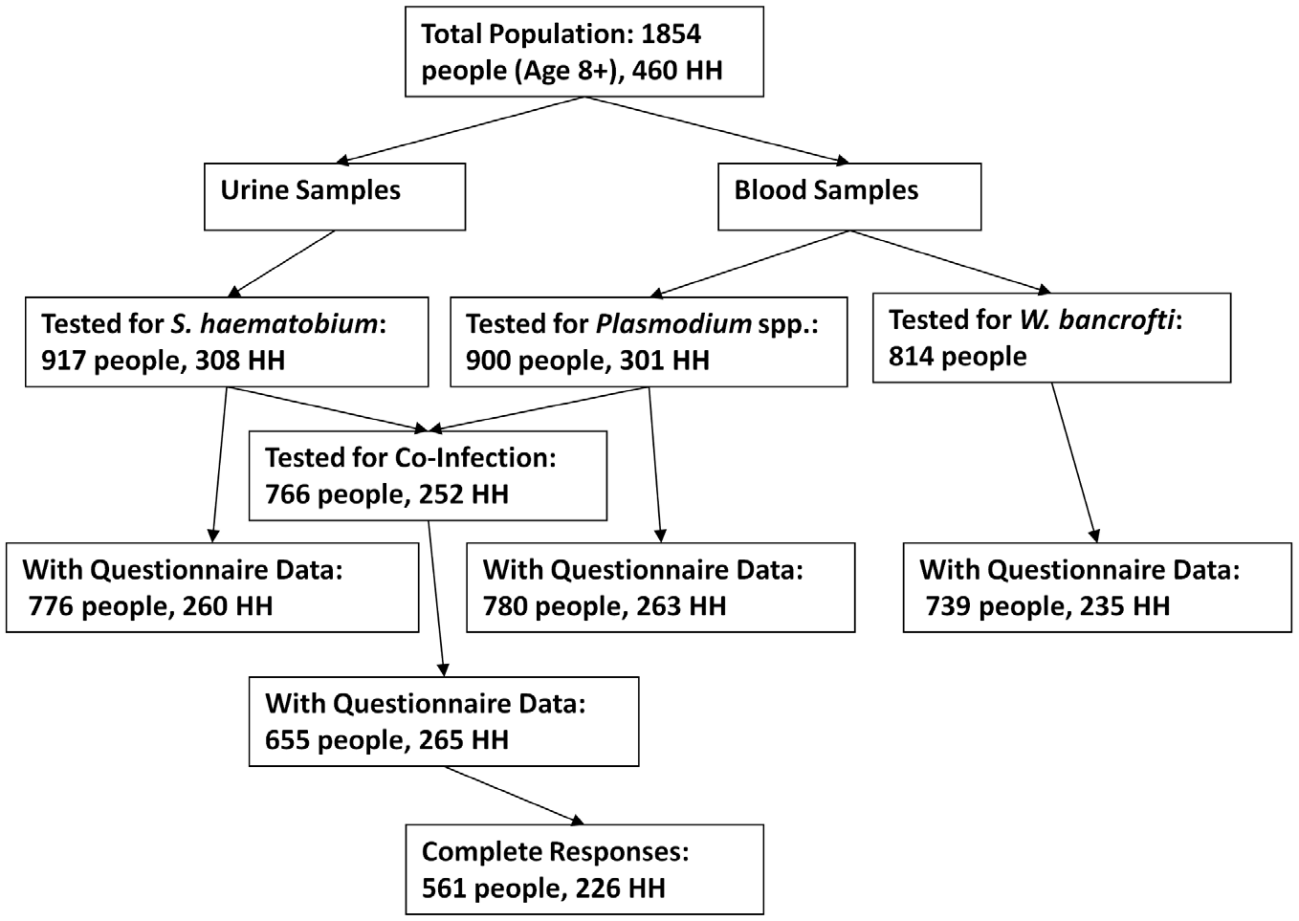
Selection of study population. A schema of the selection process for the study: the total population of Kingwede, the number who were tested for each parasite, and the number who completed questionnaires. The final dataset was comprised of individuals tested for both *Plasmodium* spp. and *S. haematobium* with no missing questionnaire data. (Abbreviation: HH = households).

A total of 1,854 persons 8 years of age and older from 460 households was identified in the study area. Approximately half (935) of the eligible individuals, representing 310 different households, agreed to participate in the study by consenting to contribute samples and answer questions. The proportions of the eligible population contributing parasitological samples for each infection and those contributing questionnaire data varied (Figure 2). Complete parasitological and at least partial questionnaire data were received for 766 (41.3%) individuals from 252 (54.8%) households. By further restricting the dataset to those with no missing questionnaire responses for the variables needed in subsequent analyses 561 persons from 226 households remained. This study population represented the total eligible population in its age distribution but adult males were underrepresented (30.8% of participating adults were male, 47.1% of the eligible adult population was male). Non-participants were not otherwise significantly different from participants in terms of age or household size, nor were the participant households significantly clustered within the village (data not shown).

When the study population was stratified by age, characteristics of children (8 to 17 yo) differed greatly from those of adults (Table 1). Adults had higher malaria and schistosomiasis KAP scores than did children, with more adults reporting a regular income, using bednets and going outside at night. Since only a few children reported having a regular income, this variable was not used in subsequent child analyses. Children were more likely than adults to report water contact with potentially infected water sources. Recent consumption of antimalarials was similar between children and adults, as were household SEP scores and distances of households to the local stream.

**Table 1:** Age-stratified characteristics of the study population.

Social and Demographic Characteristics	Overall (n = 561)†	Children (n = 223)†	Adults (n = 338)†	Odds Ratio* (children/adults)	95% CI	p-value
Age (years)	28.7 (18.3)	12.1 (2.7)	39.6 (15.8)	–	–	–
Male	38.5	50.2	30.8	2.32	1.69–3.20	<0.0001
Bed net use	55.1	48.0	59.8	0.62	0.45–0.84	0.002
Recent antimalarial treatment	59.7	56.5	61.8	0.81	0.57–1.15	0.2325
Outdoor night activity	67.9	49.8	79.9	0.25	0.17–0.36	<0.0001
Malaria KAP (0–3)	2.5 (0.8)	2.19 (1.01)	2.77 (0.55)	0.56	0.48–0.65	<0.0001
Schistosomiasis KAP (0–3)	1.8 (1.2)	1.35 (1.22)	2.12 (1.04)	0.46	0.38–0.56	<0.0001
Water contact	41.4	49.3	36.1	1.71	1.19–2.45	0.0036
Regular income	51.8	1.79	84.9	0.003	0.001–0.01	<0.0001
Household SEP score (−1.4–4.1)	0.14 (1.05)	0.19 (1.06)	0.11 (1.04)	1.09	0.92–1.28	0.3172
Household distance to stream (km)	0.66 (0.40)	0.69 (0.37)	0.64 (0.41)	1.04	0.98–1.11	0.1957
**Infection Characteristics**						
*Plasmodium* spp. (P)	50.4	75.3	34.0	6.56	4.40–9.78	<0.0001
*S. haematobium* (S)	25.0	40.8	14.5	4.09	2.77–6.04	<0.0001
P-S Co-infection	15.7	31.8	5.0	9.28	5.29–16.27	<0.0001
Heavy *Plasmodium* spp.(in positives)	26.9	36.3	13.0	3.79	1.97–7.29	<0.0001
Heavy *S. haematobium* (in positives)	15.0	15.4	14.3	1.11	0.45–2.76	0.8249

**†:** Percent of study population reporting characteristic for dichotomous variables and mean (standard deviation) for continuous variables.

*: Odds Ratios compare the odds of each social/demographic or infection characteristic among children, as compared to corresponding odds among adults.

### Infection prevalence

This population experienced high prevalence of infection by both parasites, with over 75% of participating children carrying *Plasmodium* spp. and over 40% harboring *S. haematobium* (Table 1). Co-infection was seen in 31.8% of children. In adults, infection prevalences were somewhat lower, with 34%, 14.5% and 5% carrying *Plasmodium*, *S. haematobium* and co-infection, respectively.

**Figure 3:**
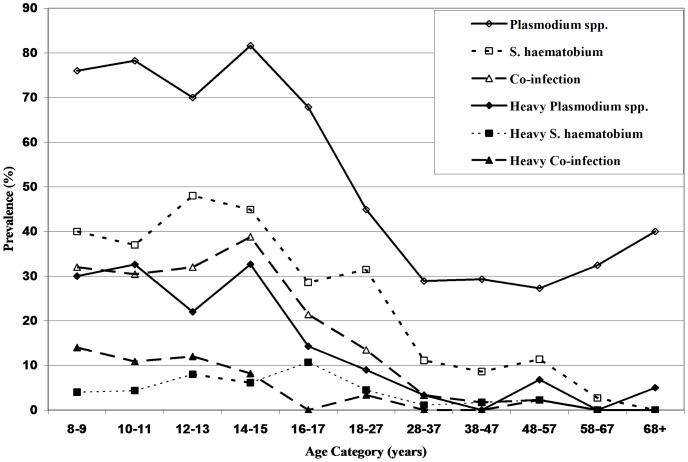
Distribution of infection prevalence and intensity by age category. The percentage of study participants (n = 561) testing positive for *Plasmodium* spp. infection, *S. haematobium* infection and co-infection. The percentage with heavy infections (as defined in the methods section) is also shown.

Children were 6.6 times more likely than adults to be infected with *Plasmodium* spp. (95% C.I. = 4.40–9.78), 4.1 times more likely to have *S. haematobium* (95% C.I. = 2.77–6.04) and 9.3 times more likely to carry *Plasmodium-S. haematobium* co-infections (95% C.I. = 5.29–16.27). Age trends in single and multiple infection prevalences are illustrated in Figure 3.

Correlates of co-infection also differed between children and adults (Tables 2 and 3). In children, the odds of co-infection with *Plasmodium* spp. and *S. haematobium* were lower for those using bednets, engaged in outdoor activities at night, and living farther from the stream, yet higher in those who reported potentially risky water contact. In adults, co-infection odds decreased with age and with increasing household SEP.

**Table 2:** Logistic GEE models of *Plasmodium* spp., *S. haematobium* and co-infection in children aged 8–17 (n = 223).

Independent Variables	*Plasmodium* spp.*	*S. haematobium* ‡	Co-infection¥
**Bed net use**			0.58 (0.32–1.03)
**Outdoor night activity**	0.55 (0.29–1.03)		**0.55 (0.31–0.99)**
**Schistosomiasis KAP**		**0.74 (0.58–0.95)**	
**Water contact**		**2.12 (1.14–3.94)**	1.71 (0.95–3.08)
**Household SEP**	0.76 (0.54–1.05)		
**Household distance to stream**		**0.36 (0.15–0.84)**	**0.32 (0.13–0.82)**

Values in the table represent odds ratios and 95% confidence intervals associated with each independent variable controlling for the other variables in the model. **Bold font** indicates a significant adjusted OR. Models were derived by stepwise elimination of the least significant correlates as identified by Wald test criteria. Original correlates also included grand mean-centered age, sex, recent malaria treatment, malaria knowledge, attitudes and practices, *S. haematobium* infection (in the *Plasmodium* spp. model), and *Plasmodium* spp. infection (in the *S. haematobium* model).

Abbreviations: KAP = knowledge, attitudes and practices; SEP = socio-economic position.

*: Referent group is children without *Plasmodium* spp. infection.

**‡:** Referent group is children without *S. haematobium* infection.

**¥:** Referent group is children with only *Plasmodium* spp. infection, with only *S. haematobium* infection or with no infection.

**Table 3:** Logistic GEE models of *Plasmodium spp.*, *S. haematobium* and co-infection in adults aged 18–86 (n = 338).

Independent Variables	*Plasmodium spp.* *	*S. haematobium* ‡	Co-infection¥
**Age** †	**0.98 (0.96–0.99)**	**0.93 (0.91–0.96)**	**0.89 (0.82–0.97)**
**Recent malaria treatment**	**0.56 (0.35–0.91)**		
**Malaria KAP**	**0.50 (0.33–0.76)**		
**Water contact**		**0.47 (0.24–0.90)**	
**Household SEP**	**0.70 (0.55–0.90)**		0.61 (0.36–1.01)

Values in the table represent odds ratios and 95% confidence intervals associated with each independent variable controlling for the other variables in the model. **Bold font** indicates a significant adjusted OR. Models were derived by stepwise elimination of the least significant correlates as identified by Wald test criteria. Original correlates also included bed net use, outdoor night activity, schistosomiasis knowledge, attitudes and practices, regular income, household distance to a stream, *S. haematobium* infection (in the *Plasmodium* spp. model), and *Plasmodium* spp. infection (in the *S. haematobium* model).

Abbreviations: KAP SEP = socio-economic position.

**†:** Grand mean centered among adults included in this analysis.

*: Referent group is adults without *Plasmodium* spp. infection.

**‡:** Referent group is adults without *S. haematobium* infection.

**¥:** Referent group is adults with only *Plasmodium* spp. infection, with only *S. haematobium* infection or with no infection.

### Infection Intensities

Among participants infected with *Plasmodium* parasites, over a quarter carried heavy infections (Table 1). Fifteen percent of participants with *S. haematobium* infections were heavily infected (100 eggs/10 mL). As with infection prevalence, the intensity of infection was much heavier in children than in adults (*Plasmodium* spp. (OR = 8.09, 95% C.I. = 4.41–14.81); *S. haematobium* OR = 3.11, 95% C.I. = 1.31–7.39). After restricting analyses to infected individuals, children were still more likely than adults to harbor heavy *Plasmodium* spp. infections (OR = 3.79, 95% C.I. = 1.97–7.29) whereas this was not the case for *S. haematobium* (Table 1).

### Co-Infection Associations

Simple regression analyses indicated that odds of *Plasmodium* spp. infection were higher in individuals infected with *S. haematobium* compared to those without (OR = 1.97, 95% C.I. = 1.34–2.89). Multivariable, multinomial logistic models confirmed this association even after adjustment for shared contextual variables among children, but this association was not seen in adults (Table 4). Children were less likely to carry single *S. haematobium* infections than to be co-infected with both parasites (OR = 0.60, 95% C.I. = 0.39–0.93). Similarly, the significant association between the intensity of *Plasmodium* spp. infection and *S. haematobium* infection observed in simple regression analyses (OR = 3.15, 95% C.I. = 1.26–7.89) persisted after adjustment for confounders in children; as noted in Table 5, children with heavy *Plasmodium* infection were more likely than those with light infection to have concomitant heavy *S. haematobium* infection, controlling for relevant covariates (OR = 1.39, 95% C.I. = 0.99–1.97). No evidence of association in odds or intensities of these infections was seen in adults (Tables 4 and 5).

**Table 4:** Multinomial odds of single infection or no infection, compared to co-infection in children and adults.

Infection Status	Children (8–17 years, n = 223)*	Adults (18+, n = 338)*
**Co-infection**	Ref	Ref
***S. haematobium*** ** only**	**0.60 (0.39–0.93)**	1.33 (0.75–2.35)
***Plasmodium*** ** spp. only**	**1.79 (1.32–2.44)**	**4.81 (3.01–7.68)**
**Neither infection**	0.83 (0.57–1.21)	**9.35 (5.96–14.66)**

Values in the table represent odds ratios (and 95% confidence intervals) for infection status other than malaria-*Schistosoma* co-infection, controlling for the other variables in the multinomial model. **Bold font** indicates a significant adjusted OR.

*: Controlling for water contact, night activity, bed net use, and household distance to water.

**Table 5:** Age-stratified adjusted odds of heavy *Plasmodium* spp. infection compared to light infections among infected individuals.

	Heavy *Plasmodium* spp.	
Independent Variables	Children (8–17 years, n = 168)	Adults (18+, n = 115)
**Bed net use**	1.75 (0.92–3.34)	
**Night activity**		**0.26 (0.08–0.93)**
**Household distance to stream**		0.27 (0.07–1.02)
***S. haematobium*** ** intensity** ‡	1.39 (0.99–1.97)	

Values within the table represent OR and 95% CI. **Bold font** indicates a significant adjusted OR. Models were derived by stepwise elimination of the least significant correlates variables as identified by Wald test criteria. Original correlates also included grand mean centered age among relevant population (children or adults), sex, recent malaria treatment, malaria knowledge, attitudes and practices, regular income (for adults model only), and household socio-economic position.

**‡:** Ordinal variable with values 0–4 representing increasing intensity. Categories based on distribution of infection intensities in the study population.

## Discussion

Results of this study reveal several key findings. Participating Kingwede residents had high prevalence of infection with *S. haematobium* and *Plasmodium* spp. parasites. Co-infection was also common and was associated with both individual- and household-level factors including young age and low socio-economic position. Interestingly, co-infection was more common than single *S. haematobium* infection in children, after controlling for other variables. Similarly, intense infections with both parasites appeared to cluster in a subset of children.

While non-participation may have biased some of estimates of association, we believe that because individual per-household participation was reasonably good (62%), our investigation illustrates the importance of context in studies of infectious diseases. We found that contextual factors (household SEP) contributed significantly to variation in an individual's parasite infection profile, including, for children, proximity to the main local water source. These findings compare with other studies of single-species infection studies in which risk of malaria and schistosomiasis was higher in households proximate to water bodies [33]–[35] and are in accord with those that associate these infections with lower SEP [36]–[39]. Our results also support recent efforts to reconsider broader social and ecological contexts in studies of infectious diseases in lieu of traditional biomedical models [40]. Finally, the identification of risk factors common to infection by both parasites argues for a broader, integrated approach to control and prevention [41] and suggests potential targets for these efforts.

Aside from contextual variables, age was found to be the most important shared risk factor of infection, with both higher prevalence and higher intensity of single and concomitant infection in school-aged children as compared to adults. The observed negative associations between age and co-infection are consistent with other published reports from endemic regions on clinical malaria [42] and *S. haematobium* infection [43]. Higher prevalence at younger ages is likely due to immunological naïveté governing susceptibility to both infections [44]–[46], a variable which was not measured in this study. The strength and consistency of the age effect on infection led to stratification of all analyses, with separate models for children and for adults. Our results support current trends in disease prevention and treatment efforts focusing on school-aged children. A key caveat may be that the many asymptomatic *Plasmodium* spp. infections in adults could be an important source for continued transmission to children and should therefore not be ignored in intervention efforts.

Age-stratified, multivariable logistic GEE analyses of *Plasmodium* spp. and *S. haematobium* prevalence revealed no evidence of positive association between two parasites for the combined *presence* of infection (measured as we did, by PCR and urine filtration) after adjustment for relevant SEP, behavioral and environmental variables (Tables 2 and 3). The apparent lack of association between these two parasite species using prevalence data has been supported by previous research [47] and could be explained by dissimilar risk factors, distributions, and modes of transmission for the two infections. Comparability of our results with those of other malaria-helminth polyparasitism studies is limited by differences in outcome measures (asymptomatic infection vs. clinical disease) [48]–[50] and helminth species of interest (most other co-infection studies investigate a variety of soil-transmitted helminth (STH) species rather than *S. haematobium*) [reviewed in 41]. However, our cross-sectional design may have limited our estimation of continuing infection risk, particularly for malaria, given annual weather related fluctuations in exposure.

In analyses of infection intensities, positive associations were observed between the *intensities* of the two parasites among co-infected children (Tables 5 and 6), thus highlighting the importance of host factors in determining parasite loads, and also suggesting a synergistic relationship. Although some studies have reported antagonistic relationships between these parasites (i.e. lower *P. falciparum* intensities in children harboring light *S. haematobium* infections vis-à-vis those not co-infected [51], [52]), many other investigations support our findings (reviewed in [53]). The potential of a synergistic relationship between these parasites is important from a clinical perspective, as well as epidemiologically. Clinically, the morbidity associated with co-infection is likely to depend on parasite loads [6], [8], [9] as seen in single-species *Plasmodium* infections [10], [11] and *S. haematobium* infections [12]. If heavy infections cluster in a subset of individuals, as suggested by our results, identification and targeting of this subset for multi-disease prevention and treatment interventions could be highly cost effective in that it would serve to reduce overall pathogen burdens.

**Table 6:** Age-stratified adjusted odds of heavy *S. haematobium* infections compared to light infections among infected individuals.

	Heavy *S. haematobium*	
Independent Variables	Children (8–17 years, n = 91)	Adults (18+ years, n = 49)
**Age** †		0.96 (0.87–1.06)
**Male**		4.19 (0.53–33.48)
**Bed net use**	**0.14 (0.03–0.61)**	–
**Schistosomiasis KAP**		0.70 (0.31–1.58)
**Water contact**		0.59 (0.09–3.98)
**Household SEP**	**1.97 (1.37–2.85)**	0.87 (0.30–2.55)
**Household distance to stream**	**0.04 (0.01–0.17)**	2.11 (0.73–6.04)
***Plasmodium*** ** spp. intensity** ‡	**1.74 (1.01–3.00)**	1.05 (0.36–3.06)

Values within the table represent OR and 95% CI. **Bold font** indicates a significant adjusted OR. Models were derived by stepwise elimination of the least significant correlates as identified by Wald test criteria. Original correlates also included recent malaria treatment and night activity (in children). Bed net use was not included in the adult model.

Abbreviations: KAP = knowledge, attitudes and practices; SEP = socio-economic position.

**†:** Grand mean centered among relevant population (children or adults) included in this analysis.

**‡:** Ordinal variable with values 0–4 representing increasing intensity. Categories based on distribution of infection intensities in the study population.

We hypothesize that immunological parameters, unmeasured in this study, are partially responsible for the observed positive association between parasite intensities. In that case, an improved understanding of the complex immunological milieu governing infection profiles would be needed to evaluate this possibility. Failure to differentiate between co-infection and co-morbidity outcomes has confounded discussion of results from other similar studies. To illustrate, data suggest that *P. falciparum* may protect against *S. haematobium* infection by promoting protective antibody development, but *Plasmodium* infection can also increase inflammatory factors associated with morbidity [54], [55].

It is also possible that seasonal or climatic variations or other unmeasured variables influenced our results. This study was cross-sectional, thus natural temporal variations in infection prevalence and intensity were not considered. Due to limited resources, we did not control for other infections such as STH or HIV that could affect susceptibility to, or intensity of *Plasmodium* spp. or *S. haematobium* infections. Concomitant infections also could have effects on host immune response, thereby influencing *S. haematobium*-*Plasmodium* spp. associations. Although STH are likely to be ubiquitous, *S. mansoni* is not endemic to this region and HIV rates are fairly low (7% prevalence in rural Kenya and 7.9% prevalence in Coast Province overall [56]), suggesting that confounding likely was minimal. Genetic factors and nutritional status represent other potentially important unmeasured confounders. Our use of marginal models should have accounted for or attenuated any genetic effects shared within families by assessing clustering of individuals within households. Furthermore, another study of *S. haematobium* from the same geographic region concluded that heritability in host susceptibility is low and unlikely to play a major role in determining individual risk for infection [57]. Potential confounding effects of nutritional status should not be large assuming similar access to nutrition within households. This also should have been partially accounted for by the inclusion of household SEP variables in analyses. Of particular note, in our study, outdoor night-time activity was associated with *lower* odds of co-infection or malaria infection among children, and with reduced odds of heavy *Plasmodium* infection in adults. This finding may be related to the endophilic feeding preference of anophelines within houses, with reduced risk among those who are out of the house for part of the night.

Future research on the epidemiology of polyparasitism could benefit from the inclusion of several key parameters not fully evaluated in this study: i) A wider range of spatial and temporal scales and study sites could reduce potential chance associations and improve understanding of the climatic, seasonal and environmental factors that influence parasite distributions and interactions; ii) Malaria investigations would greatly benefit from more sensitive, standardized diagnostic tools for quantification of species-specific *Plasmodium* parasites. Although work of other researchers has shown good correlation between median fluorescent intensity (MFI) values and parasitemia, the relationship between the two is not linear toward the upper and lower limits [28], [58], [59]. Validation of our use of MFI as a quantitative measure is needed; iii) finally, in order to fully understand the implications of observed associations between parasitic species in human hosts, a better understanding is needed of relevant immunological mechanisms [41], [53], [60]. However, the need for improved scientific knowledge about the biology, and epidemiology of polyparasitism should not take precedence over what is already known; parasites cause significant morbidity and more accessible, effective treatment and prevention is urgently required.

## References

[1] NMCP (2009). Kenyan Ministry of Health, Division of Malaria Control..

[2] King CH, Dickman K, Tisch DJ (2005). Reassessment of the cost of chronic helmintic infection: a meta-analysis of disability-related outcomes in endemic schistosomiasis.. The Lancet.

[3] Ezeamama A, McGarvey S, Acosta L, Zierler S, Manalo D (2008). The synergistic effect of concomitant schistosomiasis, hookworm and trichuris infections on children's anemia burden.. PLoS Neglected Tropical Diseases.

[4] Wilson S, Jones FM, Mwatha JK, Kimani G, Booth M (2008). Hepatosplenomegaly Is associated with low regulatory and Th2 responses to schistosome antigens in childhood schistosomiasis and malaria coinfection.. Infect Immun.

[5] Egwunyenga AO, Ajayi JA, Nmorsi OPG, Duhlinska-Popova DD (2001). *Plasmodium*/intestinal helminth co-infections among pregnant Nigerian women.. Memórias do Instituto Oswaldo Cruz.

[6] Brooker S, Akhwale W, Pullan R, Estambale B, Clarke SE (2007). Epidemiology of *Plasmodium*-helminth co-infection in Africa: Populations at risk, potential impact on anemia, and prospects for combining control.. Am J Trop Med Hyg.

[7] Brooker S, Peshu N, Warn PA, Mosobo M, Guyatt HL (1999). The epidemiology of hookworm infection and its contribution to anaemia among pre-school children on the Kenyan coast.. Transactions of the Royal Society of Tropical Medicine and Hygiene.

[8] Stephenson LS, Latham MC, Kurz KM, Kinoti SN, Oduori ML (1985). Relationships of *Schistosoma haematobium*, hookworm and malarial infections and metrifonate treatment to growth of Kenyan school children.. Am J Trop Med Hyg.

[9] Stoltzfus RJ, Chwaya HM, Tielsch JM, Schulze KJ, Albonico M (1997). Epidemiology of iron deficiency anemia in Zanzibari schoolchildren: the importance of hookworms.. Am J Clin Nutr.

[10] Mackinnon MJ, Read AF (2004). Virulence in malaria: an evolutionary viewpoint.. Philosophical Transactions of the Royal Society B: Biological Sciences.

[11] Molineaux L, Gramiccia G (1980). The Garki Project: research on the epidemiology and control of malaria in the Sudan savanna of West Africa.

[12] Warren KS, Mahmoud AAF, Muruka JF, Whittaker LR, Ouma JH (1979). Schistosomiasis haematobia in Coast Province Kenya: Relationship between egg output and morbidity.. Am J Trop Med Hyg.

[13] Bethony J, Williams JT, Kloos H, Blangero J, Alves-Fraga L (2001). Exposure to *Schistosomia mansoni* infection in a rural area in Brazil. II: Household risk factors.. Tropical Medicine and International Health.

[14] Bethony J, Williams JT, Brooker S, Gazzinelli A, Gazzinelli MF (2004). Exposure to *Schistosoma mansoni* infection in a rural area in Brazil. Part III: household aggregation of water-contact behaviour.. Tropical Medicine and International Health.

[15] Pullan R, Bethony JM, Geiger S, Cundill B, Correa-Oliveira R (2008). Human helminth co-infection: Analysis of spatial patterns and risk factors in a Brazilian community.. PLoS Neglected Tropical Diseases.

[16] Mata L (1982). Sociocultural factors in the control and prevention of parasitic diseases.. Reviews of Infectious Diseases.

[17] Brooker S, Clements ACA (2009). Spatial heterogeneity of parasite co-infection: Determinants and geostatistical prediction at regional scales.. International Journal for Parasitology.

[18] Sutherst R (2004). Global change and human vulnerability to vector-borne diseases.. Clinical Microbiology Reviews.

[19] Brooker S, Clarke SE, Njagi J, Polack S, Mugo B (2004). Spatial clustering of malaria and associated risk factors during an epidemic in a highland area of western Kenya.. Trop Med Int Health.

[20] Nacher M, Singhasivanon P, Yimsamran S, Manibunyong W, Thanyavanich N (2002). Intestinal helminths are associated with increased incidence of *Plasmodium falciparum* malaria in Thailand.. Journal of Parasitology.

[21] Nacher M, Singhasivanon P, Traore B, Vannaphan S, Gay F (2002). Helminth infections are associated with protection from cerebral malaria and increased nitrogen derivatives concentrations in Thailand.. Am J Trop Med Hyg.

[22] Spiegel A, Tall A, Raphenon G, Trape JF, Druilhe P (2003). Increased frequency of malaria attacks in subjects co-infected by intestinal worms and falciparum malaria.. Trans Roy Soc Trop Med Hyg.

[23] Bejon P, Mwangi TW, Lowe B, Peshu N, Hill AVS (2008). Helminth Infection and eosinophilia and the risk of *Plasmodium falciparum* malaria in 1- to 6-year-old children in a malaria endemic area.. PLoS Neglected Tropical Diseases.

[24] Degarege A, Animut A, Legesse M, Erko B (2009). Malaria severity status in patients with soil-transmitted helminth infections.. Acta Tropica.

[25] Peters P, Warren K, Mahmoud A (1976). Rapid, accurate quantification of schistosome eggs via nuclepore filters.. Journal of Parasitology.

[26] Mehlotra RK, Kasehagen LJ, Baisor M, Lorry K, Kazura JW (2002). Malaria infections are randomly distributed in diverse holoendemic areas of Papua New Guinea.. Am J Trop Med Hyg.

[27] McNamara DT, Thomson JM, Kasehagen LJ, Zimmerman PA (2004). Development of a multiplex PCR-Ligase detection reaction assay for diagnosis of infection by the four parasite species causing malaria in humans.. J Clin Microbiol.

[28] McNamara DT, Kasehagen LJ, Grimberg BT, Cole-Tobian J, Collins WE (2006). Diagnosing infection levels of four human malaria parasite species by a polymerase chain reaction/ligase detection reaction fluorescent microsphere-based assay.. Am J Trop Med Hyg.

[29] Warren KS, Siongok TK, Houser H, Ouma JH, Peters P (1978). Quantification of infection with *Schistosoma haematobium* in relation to epidemiology and selective population chemotherapy. I. Minimal number of daily egg counts in urine necessary to establish intenstiy of infection.. J Infect Dis.

[30] Filmer D, Pritchett LH (2001). Estimating wealth effects without expenditure data–or tears: An application to educational enrollments in States of India.. Demography.

[31] Gwatkin DR, Rustein S, Johnson K, Pande R, Wagstaff A (2000). Socio-economic differences in health, nutrition, and population in Kenya.

[32] Raghunathan TE, Solenberger PW, Hoewyk JV (2002). IVEware: Imputation and Variance Estimation Software.. Ann Arbor: Survey Methodology Program, Survey Research Center, Institute for Social Research, University of Michigan.

[33] Cano J, Descalzo M, Moreno M, Chen Z, Nzambo S (2006). Spatial variability in the density, distribution and vectorial capacity of anopheline species in a high transmission village (Equatorial Guinea).. Malaria Journal.

[34] Oesterholt M, Bousema JT, Mwerinde OK, Harris C, Lushino P (2006). Spatial and temporal variation in malaria transmission in a low endemicity area in northern Tanzania.. Malaria Journal.

[35] Booth M, Vennervald BJ, Kenty L, Butterworth AE, Kariuki H (2004). Micro-geographical variation in expsoure to *Schistosoma mansoni* and malaria, and exacerbation of splenomegaly in Kenyan school-aged children.. BMC Infectious Diseases.

[36] Malaney P, Spielman A, Sachs J (2004). The malaria gap.. American Journal of Tropical Medicine and Hygiene.

[37] TDR (2002). TDR Strategic Direction: Malaria..

[38] TDR (2002). TDR Strategic Direction: Schistosomiasis..

[39] Sachs J, Malaney P (2002). The economic and social burden of malaria.. Nature.

[40] Diez-Roux A, Aiello A (2005). Multilevel analysis of infectious diseases.. Journal of Infectious Diseases.

[41] Mwangi TW, Bethony JM, Brooker S (2006). Malaria and helminth interactions in humans: an epidemiological viewpoint.. Annals of Tropical Medicine and Parasitology.

[42] Snow RW, Omumbo JA, Lowe B, Molyneux CS, Obiero J-O (1997). Relation between severe malaria morbidity in children and level of *Plasmodium falciparum* transmission in Africa.. The Lancet.

[43] Bundy DAP, Medley GF (1992). Immuno-epidemiology of human geohelminthiasis: ecological and immunological determinants of worm burden.. Parasitology.

[44] Hviid L (2005). Naturally acquired immunity to *Plasmodium falciparum* malaria in Africa.. Acta Tropica.

[45] Baird JK (1995). Host age as a determinant of naturally acquired immunity to *Plasmodium falciparum*.. Parasitology Today.

[46] Etard J, Audibert M, Dabo A (1995). Age-acquired resistance and predisposition to reinfection with *Schistosoma haematobium* after treatment with praziquantel in Mali.. American Journal of Tropical Medicine and Hygiene.

[47] Ashford R, Craig P, Oppenheimer S (1992). Polyparasitism on the Kenya coast. 1. Prevalence, and association between parasitic infections.. Annals of Tropical Medicine and Parasitology.

[48] Shapiro AE, Tukahebwa EM, Kasten J, Clarke SE, Magnussen P (2005). Epidemiology of helminth infections and their relationship to clinical malaria in southwest Uganda.. Transactions of the Royal Society of Tropical Medicine and Hygiene.

[49] Mupfasoni D, Karibushi B, Koukounari A, Ruberanziza E, Kaberuka T (2009). Polyparasite helminth infections and their association to anaemia and undernutrition in Northern Rwanda.. PLoS Negl Trop Dis.

[50] Kabatereine NB, Brooker S, Koukounari A, Kazibwe F, Tukahebwa EM (2007). Impact of a national helminth control programme on infection and morbidity in Ugandan schoolchildren.. Bull World Health Organ.

[51] Briand V, Watier L, Le Hesran J-Y, Garcia A, Cot M (2005). Coinfection with *Plasmodium falciparum* and *Schistosoma haematobium*: protective effect of schistosomiasis on malaria in Senegalese children?. American Journal of Tropical Medicine and Hygiene.

[52] Lyke KE, Dicko A, Dabo A, Sangare L, Kone A (2005). Association of *Schistosoma haematobium* infection with protection against acute *Plasmodium falciparum* malaria in Malian children.. American Journal of Tropical Medicine and Hygiene.

[53] Druilhe P, Tall A, Sokhna C (2005). Worms can worsen malaria: toward a new means to roll back malaria?. Trends in Parasitology.

[54] Remoue F, Diallo T, Angell V, Herve M, Clercq Dd (2003). Malaria co-infection in children influences antibody response to schistosome antigens and inflammatory markers associated with morbidity.. Transactions of the Royal Society of Tropical Medicine and Hygiene.

[55] Hartgers F, Yazdanbakhsh M (2006). Co-infection of helminths and malaria: modulation of the immune responses to malaria.. Parasite Immunology.

[56] KMH (2007). Kenya AIDS Indicatory Survey 2007.

[57] King CH, Blanton RE, Muchiri E, Ouma J, Kariuki H (2004). Low heritable component of risk for infection intensity and infection-associated disease in urinary schistosomiasis among Wadigo populations in Coast Province, Kenya.. American Journal of Tropical Medicine and Hygiene.

[58] Kasehagen LJ, Mueller I, McNamara DT, Bockarie MJ, Kiniboro B (2006). Changing patterns of *Plasmodium* blood-stage infections in the the Wosera region of Papua New Guinea monitored by light microscopy and high throughput PCR diagnosis.. Am J Trop Med Hyg.

[59] Mueller I, Widmer S, Michel D, Marage S, McNamara DT (2009). High sensitivity detection of *Plasmodium* species reveals positive correlations between infections of different species, shifts in age distribution and reduced local variation in Papua New Guinea.. Malaria Journal.

[60] Douradinha B, Mota MM, Luty AJF, Sauerwein RW (2008). Cross-species immunity in malaria vaccine development: Two, three, or even four for the price of one?. Infect Immun.

